# Formation of silicon nanodots via ion beam sputtering of ultrathin gold thin film coatings on Si

**DOI:** 10.1186/1556-276X-6-403

**Published:** 2011-05-31

**Authors:** Osman El-Atwani, Sami Ortoleva, Alex Cimaroli, Jean Paul Allain

**Affiliations:** 1 School of Materials Engineering, Purdue University, West Lafayette, IN 47907, USA; 2 Birck Nanotechnology Center, Purdue University, West Lafayette, IN 47907, USA; 3 School of Electrical Engineering, Purdue University, West Lafayette, IN 47907, USA; 4 School of Nuclear Engineering, Purdue University, West Lafayette, IN 47907, USA

## Abstract

Ion beam sputtering of ultrathin film Au coatings used as a physical catalyst for self-organization of Si nanostructures has been achieved by tuning the incident particle energy. This approach holds promise as a scalable nanomanufacturing parallel processing alternative to candidate nanolithography techniques. Structures of 11- to 14-nm Si nanodots are formed with normal incidence low-energy Ar ions of 200 eV and fluences above 2 × 10^17 ^cm^-2^. *In situ *surface characterization during ion irradiation elucidates early stage ion mixing migration mechanism for nanodot self-organization. In particular, the evolution from gold film islands to the formation of ion-induced metastable gold silicide followed by pure Si nanodots formed with no need for impurity seeding.

## 

Nanostructuring of semiconductor surfaces via ion beam sputtering has been shown to yield a variety of ordered nanostructures [1-3]. While there is speculation about the mechanism of nanostructure evolution on compound semiconductors, the structuring of single-component semiconductor materials, and more specifically silicon, remains elusive. Although structuring of silicon surfaces using ion beam bombardment at normal incidence was first reported by R. Gago *et al. *[4], studies, later on, have shown that structuring of silicon dots on silicon surfaces at zero incidence angle is possible only if a certain level of impurity is available on the surface during the sputtering process [5]. Moreoever, other studies have shown that irradiating silicon surfaces with no impurity seeding results in surface smoothing at normal incidence [6,7], in contradiction to the results of R. Gago *et al. *The role of impurities, which usually comes from the ion gun and the clips holding the samples, was discussed by Ozaydin *et al. *[8,9] and Sanchez-Garcia *et al. *[10] who suggested several mechanisms on how impurity seeding can induce nanostructure formation on silicon. The formation of silicides, modification of the collision cascade, and stress generation during ion bombardment were the suggested possible impurity effects on silicon nanostructuring. In this work, we report the formation of silicon nanodots on silicon substrates via low-energy ion irradiation of ultrathin film gold coatings on Si. No impurity seeding was necessary to form Si nanostructures. The gold acted as a physical catalyst to form the structures, which was later eliminated from Si nanostructures via preferential sputtering. This process is unlike the previous studies where the impurities are kept implanted in the samples due to the continuous seeding of impurity particles from ion source grids or sample grips throughout the irradiation process.

Silicon (100) samples were prepared by cleaning silicon wafers with Piranha solution (1:1, hydrogen peroxide, sulfuric acid) and subsequent acetone, water, and alcohol baths, followed by coating with gold using an SPI sputter coater. Irradiation and the *in situ *characterization of the samples were performed in the same chamber at a pressure of 2 × 10^-8 ^Torr. Irradiation was performed with 200 eV of argon ions using a low-energy, broad beam ion source. The temperature of the silicon samples was kept at nearly room temperature with active cooling. During the irradiation process, the samples were characterized *in situ *with X-ray photoelectron spectroscopy (XPS) and ion scattering spectroscopy (ISS) at different fluences. XPS scans were performed with a source analyzer angle of 54.7°. A nonmonochromatic Mg Kα (1,245.3 eV) X-ray source was used with an anode voltage of 13.0 kV and an emission current of 15.0 mA. An ISS characterization was performed using a 1,500-eV He^+ ^and a backscattering angle of 145°. The total probing beam current was 150 nA corresponding to a maximum flux of 1.4 × 10^13 ^cm^-2 ^s^-1^.

*In situ *XPS and ISS were executed using a VG Scienta R3000 charged particle analyzer (VG Scienta, Uppsala, Sweden). *Ex situ *scanning electron microscopy (SEM) characterization of gold-coated silicon and of nanostructured silicon (after irradiation) were performed using an *ex situ *H4800 field emission SEM (Hitachi High Technologies America, Inc. Schaumburg, IL, USA). The quantification of the XPS and LEISS peaks was performed using the CasaXPS and IGOR Pro software packages, respectively. At each fluence, the relative concentration of gold using LEISS data was calculated using the following equation:(1)

where *A*_Au _and *A*_Si _are the areas under the curves of Au and Si, respectively, and *σ*_Au _and *σ*_Si _are the laboratory elastic scattering cross sections of Au and Si, respectively.

Figure 1a shows the spatial profile along a horizontal line to the sample surface with XPS core level peaks of Au 4f and Si 2p. The postirradiation data shown in Figure 1b and corresponding *ex situ *SEM images (Figure 1c,d) show the effect of the Au coating. An examination of the XPS spectrum in Figure 1b shows no sign of Au, yet the SEM images show nanopatterning only on the region where the Au was deposited. In that region, nanostructures with a diameter of roughly 11-14 nm were formed. Figure 2 shows a magnified image of the silicon dots after the irradiation process. To understand how the gold film affected the nanostructure formation, *in situ *XPS and LEISS were performed during the irradiation process on another sample fully coated with 10-nm gold. It should be noted here that while XPS is capable of probing the top 1-5 nm of the surface, LEISS probes only the first layer [11,12].

**Figure 1:**
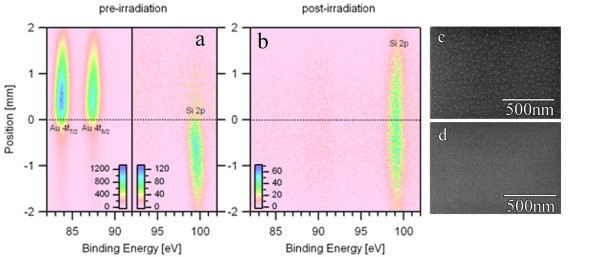
**Spatial profile of the half-coated sample before and after irradiation**. **(a) **Spatial profile of the XPS core level spectra of Au-4f and Si-2p before Ar+ 200 eV irradiation and **(b) **after irradiation. Position is plotted vertically along the sample where one region has a 20-nm Au film (top of Figure 1) and the bottom region only Si. **(c-d) **SEM images corresponding to the postirradiation condition for the Au-coated (c) and uncoated (d) regions. Si nanostructures are evidenced only in the region where Au was deposited noting that in (b) XPS Au-4f spectra are absent.

**Figure 2:**
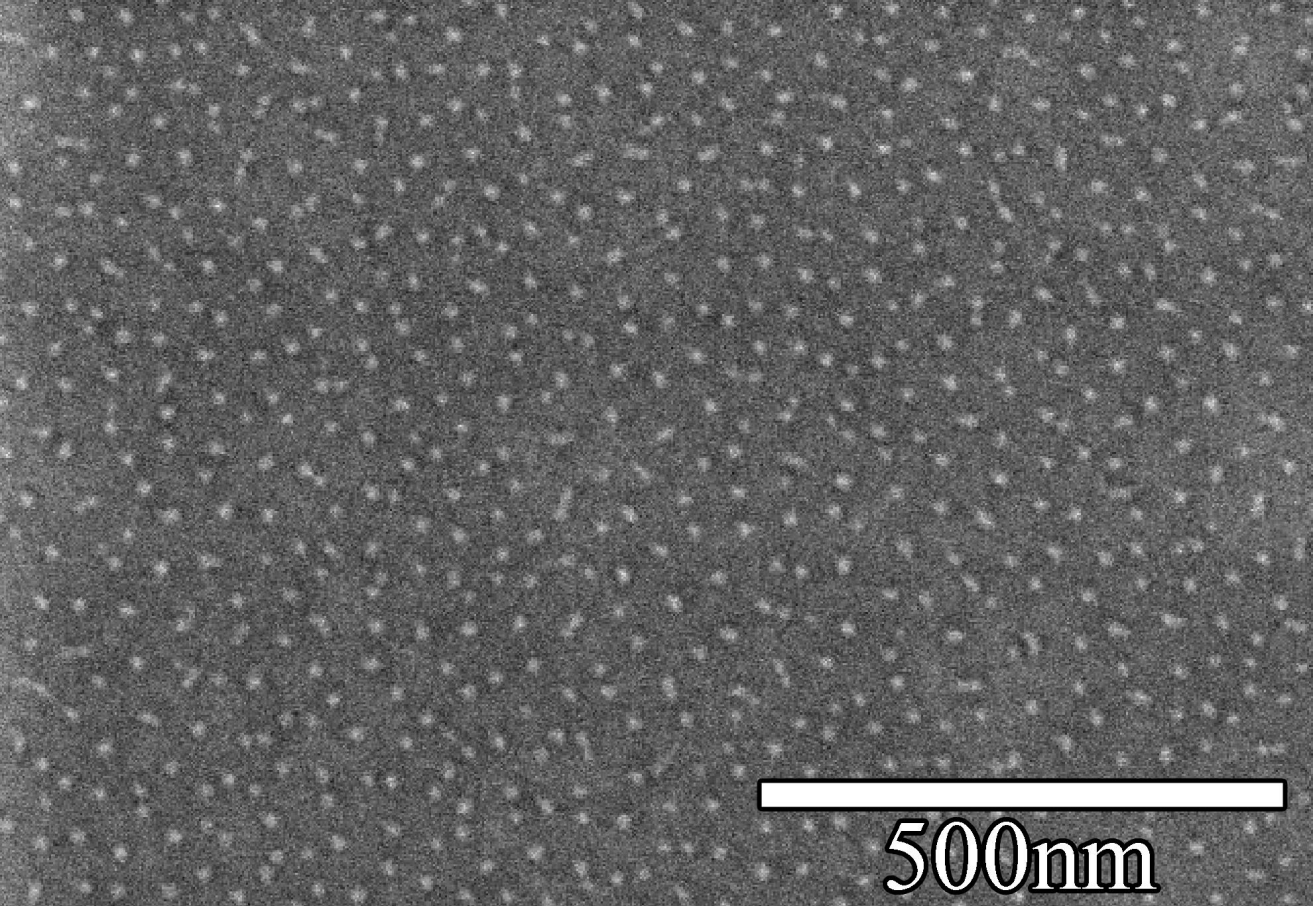
**Magnified SEM image of Silicon nanodots after the removal of the gold film**. Image was taken after a fluence of 4 × 10^17 ^cm^-2 ^after irradiation with 200 eV of Ar ions.

Figure 3a shows the *in situ *LEISS data. Before irradiation, the sample shows no silicon, and after a fluence of about 3 × 10^16 ^cm^-2^, the mixing between gold and silicon begins. Since the sputtering yield of gold is higher than silicon at 200 eV (1.13 for gold and 0.15 for silicon as calculated from the *Stopping and Range of Ions in Matter*, SRIM 2008) [13], preferential sputtering occurs until all the gold is removed, leaving silicon and a trace of oxygen on the surface top layer of the surface. The clear presence of gold in the ISS data up to a fluence of about 2.3 × 10^17 ^cm^-2 ^indicates evidence for gold-silicon mixing.

**Figure 3:**
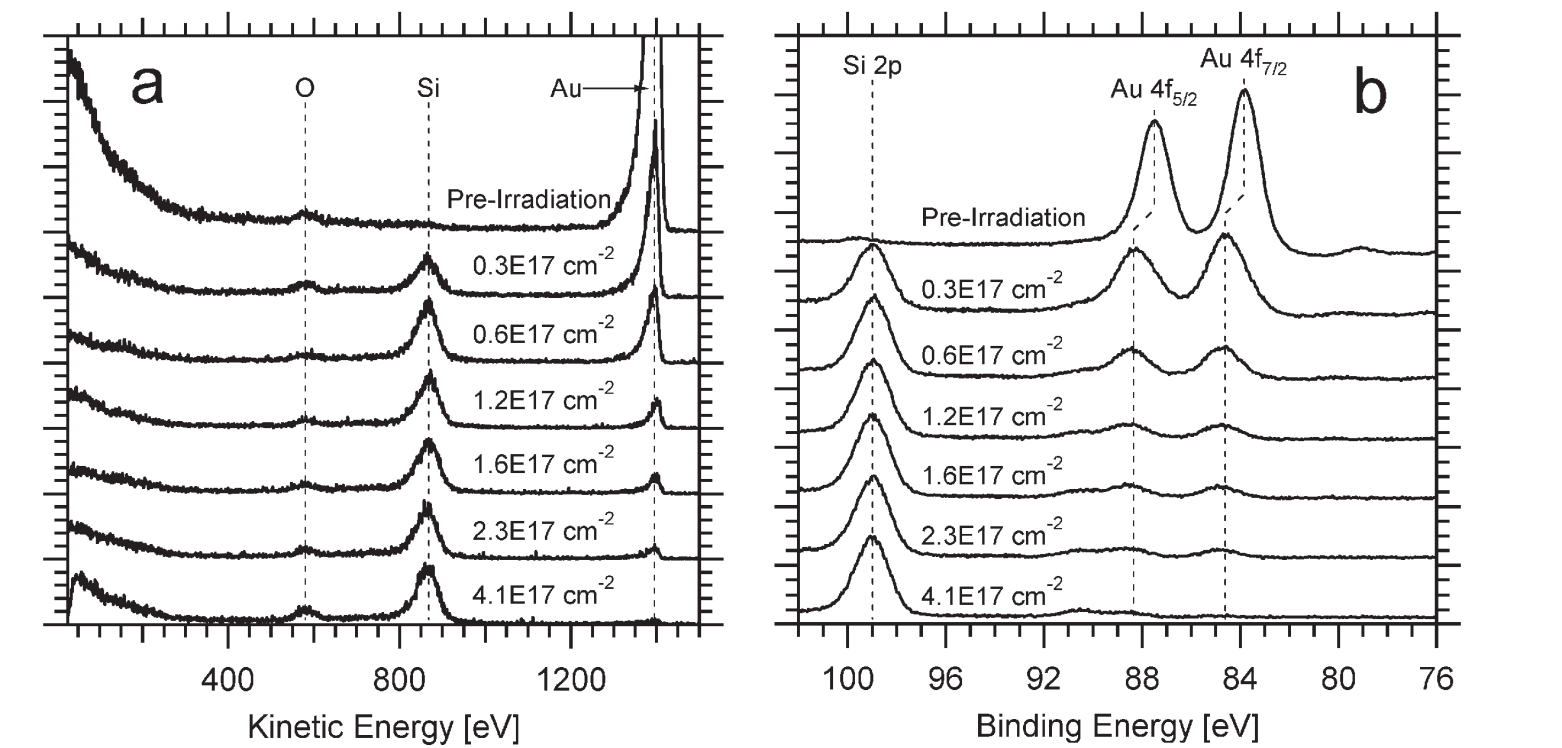
**Surface characterization of gold and silicon in the sample**. **(a) ***In situ *LEISS peaks of the three main elements on the surface of the sample (O, Si, Au). **(b) ***In situ *XPS data of gold and silicon in the sample.

The formation of gold silicides is a strong indication of the mixing between silicon and gold and has been previously discussed in the literature in the context of xenon and krypton irradiation [14,15]. Their formation is marked by a 1.0-eV shift in the XPS spectra to higher binding energies of gold after mixing; this indicates a reaction between gold and silicon [15]. Figure 3b shows the *in situ *XPS data. Gold 4f_5/2 _and 4f_7/2 _peaks were at 83.8 and 87.5 eV, respectively. After a fluence of 3 × 10^16 ^cm^-2^, the peaks shifted by 1 to 84.8 eV and 88.5 eV, respectively. This shift is due to the formation of gold silicide. The presence of the oxygen peak in the ISS and XPS data is due to the native oxide layer on top of the silicon present before coating the silicon substrates. This layer can be eliminated at higher fluences.

To elucidate about the role of gold during the nanopatterning process, a quantification of LEISS and XPS spectra was performed. The quantification results are shown in Figure 4. Both the ISS and the XPS quantification output curves indicate two different reduction mechanisms of gold concentration. Initially, gold is sputtered until the 200-eV argon ions are able to penetrate the thin gold film (penetration depth of argon is around 2 nm^10^) and induce mixing with silicon. This is marked by a large negative slope in the gold relative concentration versus fluence data shown in Figure 4, region A. The gold concentration, however, was not uniform during this period. This is due to inhomogeneities (islands) of the gold film confirmed by SEM, which during sputtering, result in more silicon areas being uncovered due to the dissimilar sputter yield of Au atoms compared to Si. Furthermore, Si and Au form a eutectic at a concentration of about 31 a/o Si-Au and temperature of 370°C. Therefore, ion-induced mixing could effectively induce an enhanced surface diffusion that redistributes Au from peak to valleys of the islands that further lead to erosion of Au. Note that when surface structures are formed, in principle, the valleys erode faster than the peaks due to the proximity of the incident particle energy deposition density to surface atoms according to the Bradley-Harper and Sigmund models [16,17].

**Figure 4:**
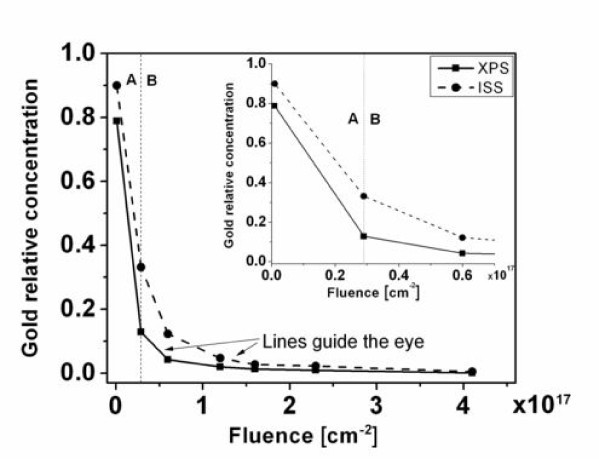
**Relative concentration of gold**. Relative concentration of gold in the sample during irradiation as a function of fluence after LEISS and XPS quantification. The plot of relative concentration (%Au) versus fluence displays two regions (A and B). Gold sputtering takes place in region A, whereas gold-silicon mixing and preferential sputtering of gold occurs in region B. The upper right inset is a magnification of the split between regions A and B.

After mixing, both gold and silicon were sputtered, and the gold relative concentration decreases much less rapidly as marked by the higher fluence tail of the exponential decay in the data (Figure 4 region B). Two regions are observed when combining the LEISS and XPS data *in situ*. Below 3 × 10^16 ^cm^-2^, since the penetration depth of Ar on Au is 2-3 nm at 200 eV, only monoelemental sputtering is the dominant erosion mechanism. However, binary collision approximation calculations show that mixing occurs at about 4 × 10^16 ^cm^-2^, very close to the experimental value (3 × 10^16^). This difference is within the relative margin of error in the ion current density measurement. After mixing ensues at 3 × 10^16 ^cm^-2^, low-energy ion scattering spectroscopy (LEISS) results indicate higher gold concentration. This is because the mixing layer thickness is less than the XPS probing depth. XPS probes the mixing layer and the silicon layers underneath, thus, is more silicon-biased. At higher fluences, however, ISS and XPS results begin to converge due to the very small amounts of gold left in the mixing layer. No impurities were found on the surface during or after the formation of the structures as revealed from the XPS and ISS data. Although the sputter yield of Au is ten times that of Si, we speculate the dominant Au concentration at the top 1-2 monolayers (along the surface nanostructures) compared to the subsurface which is likely due to the ion-induced segregation mechanism since the gold surface tension is known to be lower than Si.

After the first stage of erosion of the gold film, the second stage follows with the formation of gold silicides as indicated by the XPS data. It is well-known that gold silicide formation dominates at the bottom of the island or the Au/Si interface [18]. We conjecture that after the formation of differential silicide regions at the Au/Si interface, sputtering occurs at different rates (the silicide regions sputtering less), and thus nanostructures are effectively self-organized primarily dominated by Si. Silicides can sputter less mainly due to the enhanced binding that occurs in these cases. For example, Silicides are known to sputter about a factor of two to four times less than the pure metal component [19]. In the third and last stage at large fluences, the Au is sputtered away, and only silicon nanostructures remain.

In conclusion, silicon nanodots can be formed via low-energy ion irradiation without permanent impurity implementation. This was achieved by irradiating gold-coated silicon surfaces with argon ions at 200 eV, where gold acted as a catalyst during the nanopatterning process and was eliminated from the silicon samples after the formation of the nanodots. Silicide formation and preferential sputtering of the silicon surfaces after the gold silicide formation are the two phenomena that govern the nanodot formation process.

## Competing interests

The authors declare that they have no competing interests.

## Authors' contributions

OE and JPA planned and prepared the design of the experiment. OE, SO, and AC prepared the samples and carried out the irradiations, the LEISS and XPS characterizations. OE performed the morphology characterization with SEM. OE, SO, AC, and JPA interpreted the results and contributed to the effort of writing the manuscript.
